# Age at SARS-CoV-2 infection and psychological and physical recovery among Chinese health care workers with severe COVID-19 at 28 months after discharge: A cohort study

**DOI:** 10.3389/fpubh.2023.1086830

**Published:** 2023-02-22

**Authors:** Qian Li, Lijuan Xiong, Xiongjing Cao, Huangguo Xiong, Yanzhao Zhang, Yunzhou Fan, Liang Tang, Yang Jin, Jiahong Xia, Yu Hu

**Affiliations:** ^1^Institute of Hematology, Union Hospital, Tongji Medical College, Huazhong University of Science and Technology, Wuhan, Hubei, China; ^2^Department of Nosocomial Infection Management, Union Hospital, Tongji Medical College, Huazhong University of Science and Technology, Wuhan, Hubei, China; ^3^Department of Rehabilitation, Union Hospital, Tongji Medical College, Huazhong University of Science and Technology, Wuhan, Hubei, China; ^4^NHC Key Laboratory of Pulmonary Diseases, Department of Respiratory and Critical Care Medicine, Union Hospital, Tongji Medical College, Huazhong University of Science and Technology, Wuhan, Hubei, China; ^5^Department of Cardiovascular Surgery, Union Hospital, Tongji Medical College, Huazhong University of Science and Technology, Wuhan, Hubei, China

**Keywords:** COVID-19, cytokine, functional fitness, lymphocyte subsets, novel coronavirus

## Abstract

**Background:**

No prior study had reported the psychological and physical recovery of patients with COVID-19 2~3 years after discharge from the hospital. Moreover, it is not clear whether there is any difference in the health status of the patients with COVID-19 of different ages after discharge from the hospital.

**Methods:**

Embedding in the “Rehabilitation Care Project for Medical Staff Infected with COVID-19” in China, this study included 271 health care workers (HCWs) with severe COVID-19. Their status of health-related quality of life, persistent symptoms, functional fitness and immune function at 28 months after discharge were followed, and compared according to tertiles of age at SARS-CoV-2 infection (group of younger (≤ 33 years); medium (34-42 years); and older (≥43 years)). Multivariate linear regression and multivariable adjusted logistic regression models were applied in investigating the associations of age at SARS-CoV-2 infection and outcomes.

**Results:**

At 28 months after discharge, 76% of the HCWs with severe COVID-19 had symptom of fatigue/weakness; 18.7% of the HCWs with severe COVID-19 did not fully recover their functional fitness; the decrease of CD3^+^ T cells, CD8^+^ T cells and the increase of natural killer cells accounted for 6.6, 6.6, and 5.5%, respectively. Compared with the HCWs with severe COVID-19 in younger group, HCWs with severe COVID-19 in older group had lower scores regarding physical functioning, role physical, bodily pain and role emotional; HCWs with severe COVID-19 in older group had higher risk of cough, joint pain, hearing loss and sleep disorder; HCWs with severe COVID-19 in older group scored lower on flexibility test. The variance of relative numbers of CD3^+^ T cells, CD8^+^ T cells and natural killer cells among HCWs with severe COVID-19 of different age groups were significant.

**Conclusions:**

This study demonstrated that older HCWs with severe COVID-19 recovered slower than those with younger age regarding health-related quality of life, persistent symptoms, functional fitness and immune function at 28 months after discharge. Effective exercise interventions regarding flexibility should be performed timely to speed their rehabilitation, especially among those with older age.

## Introduction

Coronavirus Disease 2019 (COVID-19) refers to the pneumonia caused by severe acute respiratory syndrome coronavirus 2 (SARS-CoV-2) infection. The epidemic of COVID-19 has brought a huge threat to the health of people around the world. Globally, as of 5:52 pm CEST, 26 October 2022, there have been 625,740,449 confirmed cases of COVID-19, including 6,563,667 deaths, reported to World Health Organization (WHO) (1). Health care workers (HCWs) have played a very important role in the front line of the fight against the epidemic of COVID-19, but some HCWs unfortunately suffered SARS-CoV-2 infection during the battle. In China, from December 2019 to February 2020, 3019 HCWs (1,716 confirmed cases) were reported to have COVID-19 (2). Investigation among the study population with the severity of COVID-19 found that about 64% of the confirmed cases in HCWs came from Wuhan (the provincial capital of Hubei) (2). In addition, the proportion of severe cases in Wuhan (17.7%) is the highest through the whole country (2). It is of great public health significance to pay attention to the recovery of the HCWs with COVID-19, especially those with severe COVID-19.

Patients with severe COVID-19 developed dyspnea 1 week after SARS-CoV-2 infection, and severe cases rapidly progressed to acute respiratory distress syndrome, septic shock, metabolic acidosis, coagulation dysfunction, and multiple organ failure. Our (3, 4) and other prior studies (5–7) have reported on the recovery of Chinese patients with COVID-19 after discharge. Some previous studies had found that age and lifestyle behaviors were correlated with the risk of severe infection/death (8, 9) and outcome of COVID-19 infection (10, 11). Chen et al. found age was negatively linked with physical functioning (PF), role physical (RP), but positively linked with vitality (VT) in Chinese COVID-19 patients at one month follow-up (12). Iqbal et al. reported relationships of age with the presence of post-COVID-19 manifestations such as dyspnea, a persistent cough, joint pain, and chest pain in Pakistan COVID-19 recovered patients (13). Qu et al. suggested that older age was associated with poor physical component summary in Chinese HCWs with COVID-19 at the third month after discharge (14). We hypothesized that psychological and physical recovery among patients with COVID-19 after discharge may vary by age, with older patients likely to have slower recovery.

Therefore, embedding in the “Rehabilitation Care Project for Medical Staff Infected with COVID-19” in China, a prospective cohort of HCWs with COVID-19, this study aims to characterize the psychological and physical recovery among HCWs with severe COVID-19 with different age groups at SARS-CoV-2 infection in Hubei Province (the provincial capital Wuhan and its surrounding cities) at 28 months after discharge.

## Methods

### Study design and participants

The study population in this study were from “Rehabilitation Care Project for Medical Staff Infected with COVID-19” (3, 4), a prospective cohort in China. The cohort was supported by the Chinese Academy of Engineering and Tencent Charity Foundation, and was designed to follow up the health consequences (including psychological and physical) of Chinese HCWs with COVID-19 after discharge, identify high-risk population, provide timely health treatment and interventions, and promote rehabilitation for Chinese HCWs with COVID-19 after discharge.

The HCWs with COVID-19 were included in the project through the information platform of “Rehabilitation Care Project for Medical Staff Infected with COVID-19” online. Between June 2020 and March 2021, 463 severe COVID-19 were included through the platform, and they were asked to participate in follow-up visits at 28 months after discharge) in Union Hospital (Tongji Medical College, Huazhong University of Science and Technology). And 271 HCWs participated in the follow-up visits at 854.3 (844.3, 862.3) days after discharge from the hospital. More than 90% of the participants had their first symptoms between December 2019 and February 2020.

According to the recommendations by the National Health Commission (15), the severity of the COVID-19 in HCWs is categorized into 4 groups including mild, moderate, severe (meeting at least one of the following items: shortness of breath, RR ≥30 beats/min; the oxygen saturation ≤ 93%; PaO_2_/FiO_2_ ≤ 300 mmHg in the resting state), and critical (meeting at least one of the following items: respiratory failure requiring mechanical ventilation, shock, combined with other organ failure requiring intensive care unit monitoring and treatment). The specific categorizing criteria for each group are described in detail in our previous article (4). The participants with severe/critical COVID-19 were categorized into the severe group, and the participants with mild/moderate COVID-19 (with clinical symptoms, with or without pneumonia manifestations in imaging examination) were categorized into the non-severe group.

The inclusion criteria for the cohort included: (1) HCWs with severe COVID-19 in Hubei Province (the provincial capital Wuhan and its surrounding cities); (2) willing to participate in follow-up visits. In the current study, HCWs who missed follow-up visits (Short Form 36 Health Survey (SF-36) questionnaire, self-reported symptom questionnaire, functional fitness test, immune function test) at 28 months after discharge were excluded.

The enrolled HCWs with severe COVID-19 were categorized into 3 groups based on tertiles of their age at SARS-CoV-2 infection: (1) younger (≤ 33 years), (2) medium (34–42 years), and (3) older (≥43 years) group.

### Ethical aspects

This research was approved by the Ethics Committee of Union Hospital, Tongji Medical College, Huazhong University of Science and Technology (2020–0506) according to the principles of the Declaration of Helsinki. Each HCW provided informed written consent to participate in the cohort.

### Data collection

The HCWs with COVID-19 provided information on sociodemographic, lifestyle, and symptoms at admission at enrollment through the information platform of “Rehabilitation Care Project for Medical Staff Infected with COVID-19.”

At 28 months after discharge, the HCWs were invited to complete a validated SF-36 questionnaire and self-reported symptoms questionnaire through the information platform of “Rehabilitation Care Project for Medical Staff Infected with COVID-19.” The SF-36 questionnaire was developed and validated as a generic short-form instrument for measuring the health-related quality of life. The SF-36 questionnaire (Supplementary material, p. 2–4) used in this study was translated from English to Chinese, and it was proven to have good validity, reliability and internal consistency (16, 17). The SF-36 consisted of eight quality of life domains (including physical functioning (PF), RP, bodily pain (BP), general health (GH), VT, social functioning (SF), role emotional (RE) and mental health (MH)) (17) and two summary components (physical and mental components). Physical components included measurement of PF, RP, BP, and GH, and mental components included measurement of VT, SF, RE, and MH. The two components somewhat overlap or have correlations. The HCWs were required to report any new, persistent or worse symptoms than before diagnosis of COVID-19.

Between 17 June 2022 and 24 June 2022, the HCWs were invited to participate in functional fitness test under the guidance of doctors in the Department of Rehabilitation of Union Hospital (Tongji Medical College, Huazhong University of Science and Technology). As detailed described in our prior study, the Senior Fitness Test (SFT) (4) was used to assess the physical recovery status of the HCWs regarding muscle strength, flexibility and agility/dynamic balance. There are normal ranges for the scores of muscle strength and agility/dynamic balance (18, 19). As reported in our previous study, if the HCW's score in any test of muscle strength and agility/dynamic balance is below the normal ranges, the HCWs were recorded to have not recovered their functional fitness by the doctors (4).

Between 17 June 2022 and 24 June 2022, the HCWs provided blood samples in Union Hospital (Tongji Medical College, Huazhong University of Science and Technology), and the immunological indicators (cytokine profile and lymphocyte subsets) were measured at the Department of Clinical Laboratory of Union Hospital (Tongji Medical College, Huazhong University of Science and Technology) in the follow-up visits at 854.3 (844.3, 862.3) days after discharge from the hospital. The cytokine profile (IFN-γ, IL-10, IL-2 and IL-4) were measured by BD cytometric bead array analysis (the BD™ Cytometric Bead Array (CBA) Human Th1/Th2 cytokine kit). The lymphocyte subsets including relative numbers of B cells, CD3^+^ T cells, CD4^+^ T cells, CD8^+^ T cells, natural killer (NK) cells and CD4^+^/CD8^+^ cell ratio were measured by flow cytometry (BD FACSCanto™, BD Biosciences). The data of lymphocyte subsets were analyzed using FCAP software (version 3.0).

### Statistical analysis

This study used median and interquartile range (IQR) to show continuous covariates, and *n* (%) or n/N (%) to illustrate categorical covariates. χ^2^ tests were used to compare categorical variables (demographic/clinical characteristics and distribution of cytokines/lymphocyte subsets) across different age groups, and one-way analysis of variance was used to compare continuous variables (demographic characteristics and levels of cytokines/lymphocyte subsets) across different age groups. Multivariate linear regression models were applied in investigating the associations of health-related quality of life and functional fitness test with age at SARS-CoV-2 infection. Risk ratios (RRs) and 95% confidence intervals (CIs) were calculated in exploring the associations of age at SARS-CoV-2 infection and self-reported symptoms in multivariable adjusted logistic regression models. The models adjusted for sex, education status, roles in work, location of the hospital work for, smoking habit, comorbidities and body mass index (BMI). The data in this study were analyzed by SAS version 9.4 (SAS Institute, Cary, NC), and a two-sided *P* < 0.05 was considered to be statistically significant.

## Results

### Characteristics of the HCWs

Of the 463 HCWs with severe COVID-19 in “Rehabilitation Care Project for Medical Staff Infected with COVID-19” in China, 271 HCWs participated in the follow-up visits at 854.3 (844.3, 862.3) days after discharge from the hospital. In this study, 262 HCWs with severe COVID-19 completed the SF-36 questionnaire, 254 HCWs with severe COVID-19 completed the self-reported symptom questionnaire, 182 HCWs with severe COVID-19 completed the functional fitness test, and 271 completed the determination of immune indicators (cytokine profile and lymphocyte subsets) (Supplementary Figure S1).

Information on sociodemographic and clinical characteristics of the 271 HCWs with severe COVID-19 included in this study according to age at SARS-CoV-2 infection is shown in Table 1. The participants were categorized into 3 groups based on tertiles of age at SARS-CoV-2 infection. There were 87, 87, and 97 HCWs with severe COVID-19 in the group of younger (≤ 33 years at SARS-CoV-2 infection), medium (34–42 years at SARS-CoV-2 infection), and older (≥43 years at SARS-CoV-2 infection), respectively. The median value of BMI of the HCWs with severe COVID-19 was 23.4 (21.2–25.6) kg/m^2^. In this study, 81% (219/271) of the HCWs with severe COVID-19 were female, 75% (196/260) had college degree or above, more than half (58%, 157/271) were nurses, and 4% had the habit of smoking. There were statistically significant differences in the educational background, the location of the hospital work for, and the roles in work among different age groups (Table 1).

**Table 1:** Characteristics of HCWs with severe COVID-19 according to age at SARS-CoV-2 infection (*N* = 271).

**Characteristics**	**All (*N* = 271)**	**Age at SARS-CoV-2 infection (years)**			
		**Younger (** ≤ **33**, ***n*** = **87)**	**Medium (34–42**, ***n*** = **87)**	**Older (**≥**43**, ***n*** = **97)**	***P***
**Demographic characteristics**					
Age at SARS-CoV-2 infection (years)	38.0 (31.5–48.0)	30.0 (27.0–31.0)	38.0 (36.0–40.0)	49.0 (47.0–52.0)	< 0.001^*^
BMI (kg/m^2^)	23.4 (21.2–25.6)	23.1 (20.5–25.2)	23.0 (21.1–25.4)	22.8 (20.8–25.2)	0.525
Sex (female)	219 (81%)	74 (85%)	68 (78%)	77 (79%)	0.464
Education (College and higher)	196/260 (75%)	74/86 (86%)	76/85 (89%)	46/87 (53%)	< 0.001^*^
Location of the hospital work for					0.002^*^
Hankou, Wuhan	141 (52%)	52 (60%)	43 (49%)	46 (47%)	
Wuchang, Wuhan	85 (31%)	14 (16%)	30 (35%)	41 (42%)	
Hanyang, Wuhan	17 (6%)	6 (7%)	8 (9%)	3 (3%)	
Outside Wuhan in Hubei	28 (10%)	15 (17%)	6 (7%)	7 (7%)	
Roles in work					< 0.001^*^
Doctors	61 (23%)	16 (18%)	26 (30%)	19 (20%)	
Nurses	157 (58%)	64 (74%)	51 (59%)	42 (43%)	
Other	53 (20%)	7 (8%)	10 (12%)	36 (37%)	
Smoke habit (yes)	11/269 (4%)	2/86 (2%)	2 (2%)	7/96 (7%)	0.302
**Clinical characteristics**					
Comorbidities (yes)	86/267 (32%)	22/86 (26%)	29/86 (34%)	35/95 (37%)	0.539
**Symptoms at admission**					
Fatigue	177 (65%)	55 (63%)	53 (61%)	69 (71%)	0.307
Fever	166 (61%)	51 (59%)	57 (66%)	58 (60%)	0.604
Muscle soreness	114 (42%)	28 (32%)	42 (48%)	44 (45%)	0.071
Chest distress	98 (36%)	22 (25%)	35 (40%)	41 (42%)	0.036^*^
Dry cough	96 (35%)	28 (32%)	26 (30%)	42 (43%)	0.123
Cough	95 (35%)	29 (33%)	32 (37%)	34 (35%)	0.893
Shortness of breath	93 (34%)	25 (29%)	30 (34%)	38 (39%)	0.330
Diarrhea	75 (28%)	25 (29%)	20 (23%)	30 (31%)	0.469
Headache	72 (27%)	25 (29%)	15 (17%)	32 (33%)	0.046^*^
Dyspnoea	53 (20%)	16 (18%)	18 (21%)	19 (20%)	0.930
Vomiting	20 (7%)	7 (8%)	3 (3%)	10 (10%)	0.198

Data are presented as n (%), n/N (%), or median (IQR). BMI, body mass index; HCWs, health care workers; IQR, interquartile range.

^*^P < 0.05.

### Age at SARS-CoV-2 infection and health-related quality of life

**Figure 1:**
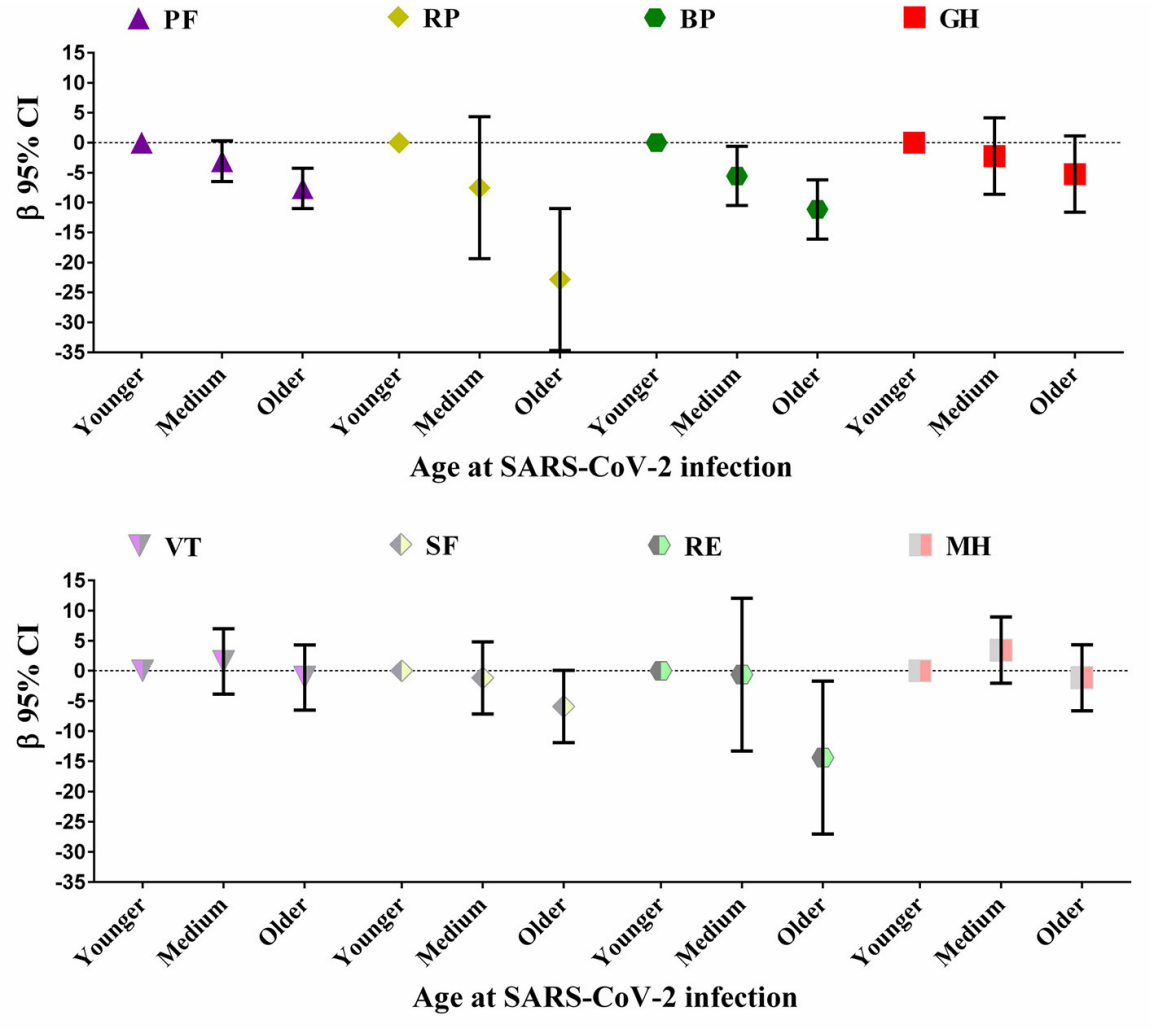
Multivariate linear regression of age at SARS-CoV-2 infection and health-related quality of life at 28 months after discharge among HCWs with severe COVID-19 (*N* = 262). BP, bodily pain; CI, Confidence interval; GH, general health; MH, mental health; PF, physical functioning; RE, role emotional; RP, role physical; SF, social functioning; VT, energy/vitality. The models adjusted for sex, education status, roles in work, location of the hospital work for, smoking habit, comorbidities and body mass index (BMI).

At the 28-month follow-up after discharge, 262 HCWs with severe COVID-19 completed measurement of the health-related quality of life using SF-36 questionnaire. The results of the linear regression models showed that, after adjusting for variables of sex, education status, roles in work, location of the hospital work for, smoking habit, comorbidities and BMI, the HCWs with severe COVID-19 with older age at SARS-CoV-2 infection (≥43 years) had lower scores regarding PF (β −7.64 [−11.02, −4.26]), RP (β −22.85 [−36.68, −11.02]), BP (β −11.14 [−16.10, −6.18]), and RE (β −14.38 [−27.04, −1.72]) compared with those with younger age (≤ 33 years) (Figure 1). There was no significant difference in the scores of GH, VT, SF and MH according to different age groups.

### Age at SARS-CoV-2 infection and persistent symptoms

A total of 254 HCWs with severe COVID-19 completed the persistent symptom questionnaire at 28 months after discharge. The top 3 residual symptoms were fatigue/weakness (76%, 194/254), palpitations (42%, 106/254) and shortness of breath (41%, 103/254). Compared with the HCWs with severe COVID-19 in younger group, the HCWs with severe COVID-19 in older group had higher risk of cough (RR 3.00 [1.09, 8.30]). The top 4 somatization symptoms were decreased exercise endurance (62%, 157/254), memory impairment (48%, 122/254), joint pain (34%, 87/254) and hearing loss (20%, 50/254). The HCWs with severe COVID-19 in older group had higher risk of developing joint pain (RR 2.83 [1.23, 6.52]) and hearing loss (RR 4.70 [1.62, 13.68]) than those in younger group. The top 3 psychological symptoms were sleep disorder, tiredness and anxiety, and the risk of sleep disorder in the older group was approximately double that of the younger group (RR 2.16 [1.01, 4.60]) (Table 2).

**Table 2:** Persistent symptoms at 28 months after discharge among HCWs with severe COVID-19 according to age at SARS-CoV-2 infection (*N* = 254).

**Categories**	**Total (*N* = 254)**	**Age at SARS-CoV-2 infection (years)**			**RR (95%CI)**		
		**Younger (** ≤ **33**, ***n*** = **80)**	**Medium (34–42**, ***n*** = **85)**	**Older (**≥**43**, ***n*** = **89)**	**Younger (** ≤ **33**, ***n*** = **80)**	**Medium (34–42**, ***n*** = **85)**	**Older (**≥**43**, ***n*** = **89)**
**Residual symptoms**							
Fatigue/weakness	194 (76%)	56 (70%)	68 (80%)	70 (79%)	1	1.95 (0.89, 4.29)	2.14 (0.89, 5.17)
*P*						0.096	0.090
Palpitations	106 (42%)	30 (38%)	37 (44%)	39 (44%)	1	1.30 (0.66, 2.57)	1.26 (0.60, 2.63)
*P*						0.451	0.545
Shortness of breath	103 (41%)	34 (43%)	29 (34%)	40 (45%)	1	0.74 (0.36, 1.51)	1.03 (0.48, 2.23)
*P*						0.404	0.934
Hair loss	85 (33%)	28 (35%)	27 (32%)	30 (34%)	1	0.89 (0.44, 1.78)	0.92 (0.43, 1.97)
*P*						0.733	0.829
Dizziness	65 (26%)	19 (24%)	17 (20%)	29 (33%)	1	0.74 (0.32, 1.69)	1.25 (0.54, 2.92)
*P*						0.473	0.602
Difficulty breathing	48(19%)	21 (26%)	14 (16%)	12 (13%)	1	0.53 (0.23, 1.23)	0.41 (0.16, 1.05)
*P*						0.141	0.064
Cough	42 (17%)	9 (11%)	11 (13%)	22 (25%)	1	1.32 (0.48, 3.63)	3.00 (1.09, 8.30)^*^
*P*						0.596	0.034^*^
Bloating	25 (10%)	5 (6%)	5 (6%)	15 (17%)	1	0.74 (0.19, 2.97)	1.81 (0.50, 6.48)
*P*						0.673	0.363
Diarrhea	17 (7%)	7 (9%)	3 (4%)	7 (8%)	NA	NA	NA
Intermittent heat	13 (5%)	3 (4%)	3 (4%)	7 (8%)	NA	NA	NA
Nausea	11 (4%)	3 (4%)	4 (5%)	4 (8%)	NA	NA	NA
Runny nose	8 (3%)	3 (4%)	1 (1%)	4 (4%)	NA	NA	NA
**Somatization symptoms**							
Decreased exercise endurance	157 (62%)	50 (63%)	47 (55%)	60 (67%)	1	0.80 (0.41, 1.58)	1.36 (0.63, 2.93)
*P*						0.522	0.431
Memory impairment	122 (48%)	33 (41%)	39 (46%)	50 (56%)	1	1.17 (0.60, 2.26)	1.42 (0.69, 2.91)
*P*						0.644	0.337
Joint pain	87 (34%)	18 (23%)	27 (32%)	42 (47%)	1	2.08 (0.93, 4.65)	2.83 (1.23, 6.52)^*^
*P*						0.075	0.014^*^
Hearing loss	50 (20%)	7 (9%)	17 (20%)	26 (29%)	1	2.97 (1.05, 8.35)^*^	4.70 (1.62, 13.68)^*^
*P*						0.04^*^	0.005^*^
Muscle pain	47 (19%)	11 (14%)	12 (14%)	24 (27%)	1	1.00 (0.38, 2.65)	1.45 (0.56, 3.80)
*P*						0.995	0.445
Limited activity	47 (19%)	13 (16%)	16 (19%)	18 (20%)	1	1.47 (0.61, 3.53)	1.25 (0.49, 3.16)
*P*						0.386	0.645
Hyposmia	40 (16%)	10 (13%)	14 (16%)	16 (18%)	1	1.43 (0.55, 3.73)	1.01 (0.36, 2.83)
*P*						0.465	0.989
**Psychological symptoms**							
Sleep disorder	119 (47%)	29 (36%)	29 (34%)	51 (57%)	1	1.55 (0.78, 3.07)	2.16 (1.01, 4.60)^*^
*P*						0.210	0.045^*^
Tiredness	115 (45%)	32 (40%)	40 (47%)	43 (48%)	1	1.28 (0.66, 2.49)	1.49 (0.73, 3.06)
*P*						0.462	0.278
Anxiety	105 (41%)	33 (41%)	31 (36%)	41 (46%)	1	0.75 (0.38, 1.48)	1.21 (0.59, 2.51)
*P*						0.414	0.603
Irritability	62 (24%)	16 (20%)	23 (27%)	23 (26%)	1	1.96 (0.88, 4.34)	2.04 (0.85, 4.89)
*P*						0.098	0.111
Uneasy	54 (21%)	15 (19%)	15 (18%)	24 (27%)	1	0.98 (0.42, 2.32)	1.95 (0.81, 4.71)
*P*						0.970	0.137
Tension	50 (20%)	19 (24%)	10 (12%)	21 (24%)	1	0.42 (0.17, 1.04)	1.09 (0.45, 2.62)
*P*						0.062	0.849
Loss of interest	39 (15%)	10 (23%)	12 (14%)	17 (19%)	1	1.10 (0.42, 2.90)	1.83 (0.66, 5.06)
*P*						0.850	0.248
Loss of appetite	28 (11%)	12 (15%)	9 (11%)	7 (8%)	1	0.59 (0.20, 1.70)	0.37 (0.10, 1.37)
*P*						0.327	0.137
Lethargy	26 (10%)	9 (11%)	8 (9%)	9 (10%)	1	0.86 (0.30, 2.52)	0.98 (0.31, 3.11)
*P*						0.788	0.978

CI, Confidence interval; HCWs, health care workers; NA, not applicable; RR, risk ratio. Data are n (%). ^*^P < 0.05. The models adjusted for variables of sex, education, roles in work, location of the hospital work for, smoke habit, comorbidities and BMI.

### Age at SARS-CoV-2 infection and functional fitness test

A total of 182 HCWs with severe COVID-19 completed functional fitness test at 28 months after discharge. In this study, 18.7% (34/182) of the HCWs with severe COVID-19 did not fully recover their functional fitness, and 16.1% (9/56), 20.6% (13/63) and 19.0% (12/63) of the HCWs with severe COVID-19 in the younger group, the medium group and the older group did not recover their functional fitness, respectively, but the difference was not statistically significant significance. This study found that the HCWs with severe COVID-19 in older group scored lower on flexibility test than those in younger group (back scratch test (left), β −3.82 [−6.41, −1.23]; back scratch test (right), β −3.28 [−5.56, −1.00]) (Table 3). There were no statistically significant differences in scores of muscle strength test and agility/dynamic balance among the three age groups.

**Table 3:** Functional fitness at 28 months after discharge among HCWs with severe COVID-19 according to age at SARS-CoV-2 infection (*N* = 182).

**Categories**	**Total (*N* = 182)**	**Age at SARS-CoV-2 infection (years)**			β **(95%CI)**		
		**Younger (** ≤ **33**, ***n*** = **56)**	**Medium (34–42**, ***n*** = **63)**	**Older (**≥**43**, ***n*** = **63)**	**Younger (** ≤ **33**, ***n*** = **56)**	**Medium (34–42**, ***n*** = **63)**	**Older (**≥**43**, ***n*** = **63)**
**Muscle strength test**							
Grip strength test, *N*	28.9 (24.9, 33.1)	29.2 (24.5, 31.7)	29.6 (26.2, 34.0)	27.4 (24.5, 32.9)	0	−0.04 (−2.27, 2.20)	−0.49 (−2.72, 1.75)
30-s elbow flexion test, *n*	21.0 (18.0, 25.0)	20.0 (17.0, 23.0)	22.0 (19.0, 24.8)	21.0 (18.0, 25.0)	0	0.73 (−1.03, 2.49)	0.35 (−1.41, 2.10)
30-s chair stand, *n*	18.0 (16.0, 220)	17.5 (16.0, 21.0)	19.0 (16.0, 22.8)	18.0 (15.0, 22.0)	0	0.73 (−1.18, 2.64)	−0.41 (−2.32, 1.51)
2-min step test, *n*	93.0 (81.0, 107.0)	93.5 (85.0, 103.5)	93.0 (79.3, 106.5)	93.0 (84.0, 110.0)	0	−3.08 (−11.26, 5.11)	0.82 (−7.40, 9.05)
**Flexibility test**							
Back scratch test (left)	1.0 (−57, 2.5)	1.9 (−2.1, 3.1)	1.5 (−4.0, 2.5)	−3.0 (−8.8, 1.5)	0	−0.48 (−3.09, 2.13)	−3.82 (−6.41, −1.23)^*^
Back scratch test (right)	2.0 (−2.0, 3.8)	2.8 (0.0, 5.0)	2.4 (0.2, 4.0)	0.5 (−7.3, 2.3)	0	0.44 (−1.86, 2.74)	−3.28 (−5.56, −1.00)^*^
Chair sit-and-reach test, cm	2.4 (−0.4, 5.4)	2.6 (0.6, 5.9)	2.3 (−0.4, 5.6)	1.8 (−1.7, 4.1)	0	0.40 (−1.92, 2.72)	−0.44 (−2.76, 1.88)
**Agility/dynamic balance**							
Functional reach test, cm	26.4 (21.7, 30.7)	26.8 (23.1, 31.7)	26.7 (21.7, 30.3)	25.6 (20.6, 30.4)	0	−1.52 (−3.92, 0.89)	−1.88 (−4.29, 0.53)
**YBT**							
Anterior-L^‡^	74.0 (68.9, 79.3)	74.4 (69.7, 78.8)	73.9 (69.9, 80.6)	74.3 (66.8, 79.3)	0	−1.29 (−4.52, 1.95)	−1.68 (−4.93, 1.58)
Posterolateral-L	80.9 (73.0, 88.2)	82.0 (75.2, 89.1)	81.2 (73.4, 86.9)	79.5 (71.0, 87.0)	0	−1.87 (−5.55, 1.81)	−3.27 (−6.96, 0.43)
Posteromedial-L	67.3 (59.9, 75.4)	68.3 (61.6, 76.6)	68.3 (61.1, 73.8)	66.3 (54.9, 75.1)	0	−2.76 (−7.48, 1.97)	−3.93 (−8.70, 0.84)
Anterior-R^†^	74.7 (69.0, 80.3)	75.1 (69.3, 80.2)	74.2 (70.3, 80.1)	74.7 (66.9, 80.8)	0	−1.31 (−4.93, 2.33)	−1.75 (−5.38, 1.88)
Posterolateral-R	81.2 (74.3, 88.2)	84.5 (77.0, 87.5)	80.8 (74.4, 88.4)	80.1 (72.0, 88.0)	0	−1.85 (−6.18, 2.49)	−2.55 (−6.89, 1.78)
Posteromedial-R	69.1 (61.0, 78.1)	72.2 (64.0, 77.9)	67.8 (61.0, 78.0)	68.0 (59.0, 79.0)	0	−2.89 (−7.21, 1.44)	−1.91 (−6.26, 2.43)

HCWs, health care workers; IQR, interquartile range; ^‡^L-reach distance by left leg.^†^R-reach distance by right leg. ^*^p < 0.05.

The models adjusted for sex, education, roles in work, location of the hospital work for, smoke habit, comorbidities and BMI.

### Age at SARS-CoV-2 infection and immune function test

From June 17, 2022 to June 24, 2022, 271 HCWs with severe COVID-19 participated in the follow-up visits at 854.3 (844.3, 862.3) days after discharge from the hospital. As shown in Table 4, more than 98% of the HCWs with severe COVID-19 had cytokine levels (IFN-γ, IL-10, IL-2 and IL-4) in the normal ranges. There were statistically significant differences in the levels of IL-10 cytokines among HCWs with severe COVID-19 in different age groups. The levels and distribution of lymphocyte subsets of the HCWs with severe COVID-19 are shown in Table 5, and it was found that the relative numbers of CD3^+^ T cells, CD8^+^ T cells and NK cells among HCWs with severe COVID-19 of different age groups were different. The decrease of CD3^+^ T cells, CD8^+^ T cells and the increase of NK cells accounted for 6.6, 6.6 and 5.5%, respectively. In the HCWs with severe COVID-19 of older group, the decrease of CD3^+^ T cells, CD8^+^ T cells and the increase of NK cells accounted for 12.4, 14.4, and 10.3%, respectively.

**Table 4:** Levels and distribution of cytokines at 28 months after discharge among HCWs with severe COVID-19 according to age at SARS-CoV-2 infection (*N* = 271).

**Categories**	**Total (*N* = 271)**	**Age at SARS-CoV-2 infection (years)**			***P***
		**Younger (** ≤ **33**, ***n*** = **87)**	**Medium (34–42**, ***n*** = **87)**	**Older (**≥**43**, ***n*** = **97)**	
**IFN-γ** **(pg/mL)**	1.50 (1.33–1.71)	1.56 (1.41–1.74)	1.48 (1.31–1.73)	1.50 (1.33–1.66)	0.480
Elevated	1 (0.04%)	0 (0.0%)	1 (1.1%)	0 (0.0%)	
Normal^†^	0.71–4.17	0.98–4.17	0.71–2.17	0.91–2.25	
Decreased	0 (0.0%)	0 (0.0%)	0 (0.0%)	0 (0.0%)	
**IL-10 (pg/mL)**	2.12 (1.73–2.58)	2.12 (1.81–2.48)	1.99 (1.47–2.39)	2.25 (1.81–2.78)	0.012^*^
Elevated	1 (0.04%)	0 (0.0%)	0 (0.0%)	1 (1.0%)	
Normal^†^	0.59–4.49	0.59–4.49	0.94–3.74	0.71–4.15	
Decreased	0 (0.0%)	0 (0.0%)	0 (0.0%)	0 (0.0%)	
**IL-2 (pg/mL)**	2.42 (2.16–2.77)	2.42 (2.11–2.67)	2.47 (2.21–2.77)	2.42 (2.13–2.67)	0.469
Elevated	0 (0.0%)	0 (0.0%)	0 (0.0%)	0 (0.0%)	
Normal^†^	1.55–3.56	1.79–3.56	1.55–3.36	1.74–3.56	
Decreased	0 (0.0%)	0 (0.0%)	0 (0.0%)	0 (0.0%)	
**IL-4 (pg/mL)**	1.84 (1.60–2.14)	1.84 (1.60–2.09)	1.94 (1.70–2.19)	1.84 (1.54–2.14)	0.584
Elevated	2 (0.74%)	1 (1.1%)	0 (0.0%)	1 (1.0%)	
Normal^†^	0.49–3.16	0.59–3.06	1.10–3.06	0.49–3.16	
Decreased	0 (0.0%)	0 (0.0%)	0 (0.0%)	0 (0.0%)	

Data are presented as median (IQR) or n (%). ^†^Data are shown as the normal ranges of the indicators. HCWs, health care workers. ^*^P < 0.05.

**Table 5:** Levels and distribution of lymphocyte subsets at 28 months after discharge among HCWs with severe COVID-19 according to age at SARS-CoV-2 infection (*N* = 271).

**Categories**	**Total (*N* = 271)**	**Age at SARS-CoV-2 infection (years)**			***P***
		**Younger (** ≤ **33**, ***n*** = **87)**	**Medium (34–42**, ***n*** = **87)**	**Older (**≥**43**, ***n*** = **97)**	
**B cells (%)**	11.26 (8.25–14.35)	11.97 (8.37–14.19)	11.28 (8.55–14.74)	10.78 (7.48–14.31)	0.601
Elevated	22 (8.1%)	7 (8.0%)	9 (10.3%)	6 (6.2%)	0.726
Normal^†^	4.11–18.12	5.29–17.25	4.76–16.12	4.76–16.12	
Decreased	7 (2.6%)	3 (3.4%)	1 (1.1%)	3 (3.1%)	
**CD3**^**+**^ **T cells (%)**	71.54 (65.60–76.08)	73.91 (68.42–78.31)	72.41 (66.08–74.37)	69.48 (62.33–73.30)	0.001^*^
Elevated	4 (1.5%)	1 (1.1%)	0 (0.0%)	3 (3.1%)	0.019*
Normal^†^	58.29–84.15	58.47–83.76	58.54–84.15	58.54–84.15	
Decreased	18 (6.6%)	2 (2.3%)	0 (0.0%)	12 (12.4%)	
**CD4**^**+**^ **T cells (%)**	39.0 (33.76–43.43)	39.15 (35.10–42.40)	38.96 (33.42–42.46)	39.00 (33.12–44.06)	0.942
Elevated	7 (2.6%)	1 (1.1%)	2 (2.3%)	4 (4.1%)	0.593
Normal^†^	25.52–51.37	26.16–48.19	26.33–46.79	26.33–46.79	
Decreased	10 (3.7%)	3 (3.4%)	2 (2.3%)	5 (5.7%)	
**CD4**^**+**^**/CD8**^**+**^ **cell ratio**	1.68 (1.36–2.18)	1.49 (1.27–1.96)	1.62 (1.34–2.09)	1.93 (1.45–2.37)	0.001^*^
Elevated	28 (10.3%)	6 (6.9%)	7 (8.0%)	15 (15.5%)	0.113
Normal^†^	0.54–2.71	0.76–2.71	0.46–2.72	0.54–2.66	
Decreased	0 (0.0%)	0 (0.0%)	0 (0.0%)	0 (0.0%)	
**CD8**^**+**^ **T cells (%)**	22.78 (18.58–27.30)	25.65 (20.70–30.13)	22.70 (19.28–26.52)	20.56 (16.86–24.21)	< 0.001*
Elevated	3 (1.1%)	1 (1.1%)	0 (0.0%)	2 (2.1%)	0.002*
Normal^†^	14.26–38.73	14.29–38.73	22.70–38.32	14.53–37.55	
Decreased	18 (6.6%)	2 (2.3%)	2 (2.3%)	14 (14.4%)	
**NK cells (%)**	15.01 (10.91–21.62)	12.29 (9.74–18.70)	14.93 (11.55–20.26)	19.00 (13.95–23.66)	< 0.001*
Elevated	15 (5.5%)	2 (2.3%)	3 (3.4%)	10 (10.3%)	0.102
Normal^†^	3.46–29.84	3.46–28.87	3.51–30.44	4.78–27.96	
Decreased	2 (0.7%)	0 (0.0%)	1 (1.1%)	1 (1.0%)	

Data are presented as median (IQR) or n (%). ^†^Data are shown as the normal ranges of the indicators. HCWs, health care workers; NK cells, Natural killer cells. ^*^P < 0.05.

## Discussion

The hypothesis that older HCWs with severe COVID-19 recovered slower in psychological and physical aspects was completely confirmed in this study. This study is the longest follow-up time (28 months) for patients with COVID-19 after discharge worldwide, and can focus on both the psychological and physical rehabilitation of the study population. This study categorized the HCWs with severe COVID-19 according to their age at SARS-CoV-2 infection, and found for the first time that, the HCWs with severe COVID-19 in older group (≥43 years at SARS-CoV-2 infection) had higher risk of residual symptoms, somatic symptoms and psychological symptoms than those in younger group (≤ 33 years at SARS-CoV-2 infection). The HCWs with severe COVID-19 in older group scored lower on the flexibility assessment of functional fitness than those in younger group. At 28 months after discharge, cytokine levels returned to normal in almost all the study population, with 6.6%, 6.6%, and 5.5% having decreased relative numbers of CD3^+^ T cells and CD8^+^ T cells and increased relative numbers of NK cells. There were significant differences in the levels of IL-10, CD3^+^ T cells, CD8^+^ T cells, NK cells and CD4^+^/CD8^+^ cell ratio among different age groups.

### Health-related quality of life

This study found that older age at SARS-CoV-2 infection was related with worse health-related quality scores in terms of PF, RP, BP, and RE. A cross-sectional study in China also revealed that age was negatively linked with PF and RP in discharged COVID-19 patients, but it suggested positive associations between age and VT at the 1 month follow-up (12). In a multicenter study in China, Qu and colleagues found that patients of COVID-19 whose age < 60 years had higher scores of PF and GH than those whose age ≥60 years at the third month after discharge (14). A study in Vietnam showed that health-related quality scores were significantly lower in people who aged 60 years or older with suspected COVID-19 symptoms (20). Taken together, active interventions are urgently needed to help older HCWs with severe COVID-19 to improve their health-related quality of life.

### Symptoms post COVID-19

Consistent with this study, studies in Italy (21) and Egypt (22) suggested that 87.4 and 89.2% of the patients with COVID-19 had at least 1 persistent symptom (mainly fatigue) post COVID-19 manifestations. Patients infected with SARS were also found to have symptoms of fatigue for up to 4 years after discharge (23). Another Chinese study found that the main symptoms of patients with COVID-19 were fatigue/weekness and sleep difficulties at 6 months, 12 months, and 2 years after discharge (7). At 6 months, 12 months, and 2 years after discharge, 68, 49, and 55% of the participants had at least one persistent symptom, indicating persistent symptoms fluctuated or relapsed over time in the patients with COVID-19 (7). In this study, the participants were all HCWs with severe COVID-19, and this study only investigated persistent symptoms at 28 months after discharge, so it could not analyze the dynamic changes of persistent symptoms after discharge. In this study, 70%~80% of HCWs with severe COVID-19 still had symptom of fatigue/weekness at 28 months after discharge. This proportion is relatively high especially among participants with older age at SARS-CoV-2 infection. There were differences in the severity and frequency of the persistent symptoms among the participants, and it was not known whether there was overreporting. Some studies suggested that the persistent symptoms may be directly caused by the SARS-CoV-2 infection, but also related to psychological conditions (24). Therefore, effective rehabilitation programmes such as exercising (25) and psychological counseling should be carried out in time to promote their recovery. The recovery of the study population after a longer period of time should also be monitored timely.

### Functional fitness

In our previous study, 70.4, 48.9, 29.6, and 28.5% of the HCWs with severe COVID-19 did not have their functional fitness recovered at 5, 8, 11, and 13 months after discharge (3, 4). At 28 months after discharge, the proportion who did not recover dropped to 18.9%. It was clear that the functional fitness of the HCWs with severe COVID-19 gradually improved as the time to discharge increased. Flexibility mainly reflects the ranges of motions of the joints. In the symptom assessment, this study found that the HCWs with severe COVID-19 with older age were more likely to have symptom of joint pain, which is estimated to partly explain why the HCWs with severe COVID-19 with older age scored worse in terms of flexibility. Lacking of exercise can cause decreased flexibility and incorrect posture of major joints, which can lead to injury (26). Therefore, it is urgent to take rehabilitation interventions and strengthen exercise (choosing the proper type, frequency, and duration of exercise), especially to help the HCWs with severe COVID-19 with older age to improve flexibility and promote rehabilitation.

### Immunological factors

Like patients infected with SARS- CoV (27), H5N1 (28) and H7N9 (29), patients with SARS-CoV-2 (30–32) have elevated levels of cytokines in the acute and convalescent phases, suggesting suffering cytokine storms. Our and other studies reported the cytokines of the patients with COVID-19 showed a trend of decrease (indications of gradual improvement) after discharge (3, 4, 31). As reported in our prior study, at 13 months after discharge, 11.6% of the HCWs with severe COVID-19 had elevated IL-6, and more than 98% had normal levels of the remaining five cytokines (IFN-γ, IL-10, IL-2, IL-4 and TNF-α) (4). Unfortunately, in this study, IL-6 and TNF-α levels were not detected, and only normal levels of cytokines of IFN-γ, IL-10, IL-2 and IL-4 were found in more than 99% of the HCWs with severe COVID-19 at 28 months after discharge. The recovery of IL-6 needs to be monitored in follow-up studies. Mechanism studies should also be further carried out.

Consistent with our study, other studies have also found different degrees of abnormality in lymphocyte subsets after SARS-CoV-2 infection. In our previous study, we found that after discharge, the lymphocyte subsets in the HCWs with severe COVID-19 gradually became normal (3, 4). From 13 months to 28 months after discharge, the proportion of HCWs with severe COVID-19 with decreased relative numbers of CD3^+^ T cells decreased from 11.9 to 6.6%, the proportion of HCWs with severe COVID-19 with increased relative numbers of NK cells decreased from 8.6 to 5.5%, showing a trend of improvement, but the proportion of those with decreased relative numbers of CD8^+^ T cells increased slightly (3.9 vs. 6.6%). In the older group in this study, the proportion of HCWs with severe COVID-19 with abnormal lymphocyte subsets was up to 14.4%. Studies by Heo et al. also found that CD3^+^ T cells, CD4^+^ T cells, and CD8^+^ T cells decreased in men after infection with SARS-CoV-2, especially in study population in their 60s (26). Studies had found that physical exercise, such as yoga (33), can effectively increase the levels of lymphocyte subsets and improve immune function. Therefore, the monitoring of lymphocyte subsets in HCWs with severe COVID-19, especially in the older group, should be strengthened, and appropriate exercise duration, intensity and frequency should be sought to help them improve their immune function.

This study has the following limitations. First, there was a lack of a control group of survivors of respiratory infection other than SARS-CoV-2 in this study, so it is unknown whether some of the outcomes observed were caused by COVID-19 specifically. Second, the sample size of this study is relatively small (*n* = 271), although we have tried our best to include all HCWs with severe COVID-19 in Hubei Province (from December 2019 to February 2020, a total of 1474 confirmed patients and 232 with severe COVID-19 in Hubei Province) (2), which is already the largest follow-up study of HCWs with severe COVID-19 in China, and the basic characteristics of the participants included and excluded in this study are similar. Given the limited sample size, studied associations without a statistical significance should be interpreted with caution. Larger cohort studies are needed in the future. Third, The participants' symptoms were self-reported, the severity and frequency of symptoms may vary. Perhaps these symptoms are related to psychological factors of the participants, and it is not known whether these symptoms are overreported. Fourth, the increase of IL-6 is a particularly important aspect of cytokine abnormalities. In this study, four cytokines were measured. The lack of IL-6 levels made it impossible to judge the recovery of cytokines in the participants. Follow-up studies should continue to monitor. Fifth, the lymphocyte subsets in this study were measured in relative numbers, not absolute numbers. Subsequent studies should continue to report on the recovery of absolute data on lymphocyte subsets in the participants. Lastly, the participants' health status 14 days before the physical examination and status of shift work fatigue 1 day before the physical examination may affect their immune function, but we don't know the specific information. Therefore, we need to be more cautious when extrapolating our findings to other populations.

For the first time, this study reported the psychological and physical recovery of the HCWs with severe COVID-19 who were discharged from hospital for 28 months. The functional fitness and immune function of most HCWs with severe COVID-19 returned to normal at 28 months after discharge, but some of them still had persistent symptoms. Older HCWs with severe COVID-19 recovered slower than those with younger age in terms of health-related quality of life, persistent symptoms, functional fitness and immune function. Effective exercise interventions regarding flexibility should be provided for HCWs with severe COVID-19, especially for those with older age. More studies on the causes, mechanisms, rehabilitation techniques, and clinical management strategies (24, 34) for patients with COVID-19 after discharge from the hospital are needed in the future.

## Data availability statement

The original contributions presented in the study are included in the article/Supplementary material, further inquiries can be directed to the corresponding authors.

## Ethics statement

The studies involving human participants were reviewed and approved by Ethics Committee of Union Hospital, Tongji Medical College, Huazhong University of Science and Technology. The patients/participants provided their written informed consent to participate in this study.

## Author contributions

YH, JX, LX, and QL designed this study. YH, QL, and LX were responsible for the integrity of the data and the accuracy of the data analysis. LX, XC, HX, YZ, YF, and YJ collected the data. QL, LX, and LT analyzed the data. QL and YH drafted the manuscript. All authors had full access to all of the data in the study, revised the manuscript, and gave final approval for the version to be published.
