# Supplementary material for: Age at SARS-CoV-2 infection and psychological and physical recovery among Chinese health care workers with severe COVID-19 at 28 months after discharge: A cohort study
Source: Front Public Health. 2023 Feb 22;11:1086830. doi: 10.3389/fpubh.2023.1086830 (PMC9992871; doi:10.3389/fpubh.2023.1086830)
Supplement: Supplementary file 1 [file Data_Sheet_1.doc]

**Title:** Age at SARS-CoV-2 infection and psychological and physical recovery among Chinese health care workers with severe COVID-19 at 28 months after discharge: a cohort study


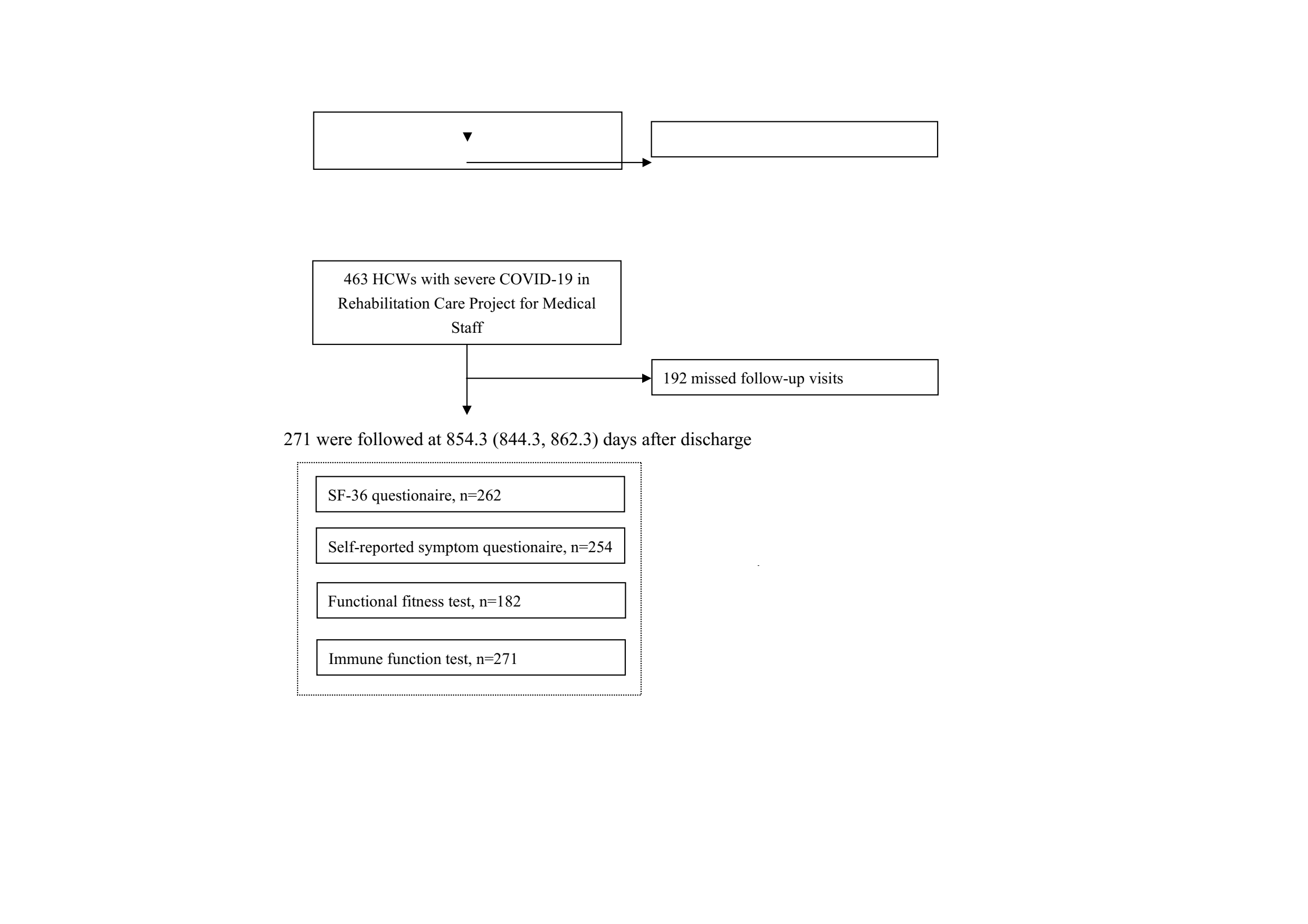
**Figure S1-Flow diagram of the study**

HCWs: health care workers

| **SF-36 QUESTIONNAIRE（SF36）** | | | |
| --- | --- | --- | --- |
| **Name：** | **Sex：** | **Age：** | **ID#：** |
| **Address：** | | | **Date：** |
| **GENERAL HEALTH:**   1. In general, would you say your health is：   ①Excellent ②Very good ③Good ④Fair ⑤Poor  2.Compared to one year ago, how would you rate your health in general now?  ①Much better now than one year ago ②Somewhat better now than one year ago ③About the same ④Somewhat worse now than one year ago ⑤Much worse than one year ago | | | |
| **LIMITATIONS OF ACTIVITIES:**  3.The following items are about activities you might do during a typical day. Does your health now limit you in these activities? If so, how much ?  （1）Vigorous activities, such as running, lifting heavy objects, participating in strenuous sports.  ①Yes, Limited a lot ②Yes, Limited a little ③No, Not limited at all   1. Moderate activities, such as moving a table, pushing a vacuum cleaner, bowling, or playing golf   ①Yes, Limited a lot ②Yes, Limited a little ③No, Not limited at all  （3）Lifting or carrying groceries  ①Yes, Limited a lot ②Yes, Limited a little ③No, Not limited at all  （4）Climbing several flights of stairs  ①Yes, Limited a lot ②Yes, Limited a little ③No, Not limited at all  （5）Climbing one flight of stairs  ①Yes, Limited a lot ②Yes, Limited a little ③No, Not limited at all  （6）Bending, kneeling, or stooping  ①Yes, Limited a lot ②Yes, Limited a little ③No, Not limited at all  （7）Walking more than 1500 m  ①Yes, Limited a lot ②Yes, Limited a little ③No, Not limited at all  （8）Walking 1000 m  ①Yes, Limited a lot ②Yes, Limited a little ③No, Not limited at all  （9）Walking 100 m  ①Yes, Limited a lot ②Yes, Limited a little ③No, Not limited at all  （10）Bathing or dressing yourself  ①Yes, Limited a lot ②Yes, Limited a little ③No, Not limited at all  **PHYSICAL HEALTH PROBLEMS:**  4、During the past 54 weeks, have you had any of the following problems with your work or other regular daily activities a result of your physical health?  （1）Cut down the amount of time you spent on work or other activities  ①Yes ②No  （2）Accomplished less than you would like  ①Yes ②No  （3）Were limited in the kind of work or other activities  ①Yes ②No  （4）Had difficulty performing the work or other activities (for example, it took extra effort)  ①Yes ②No  **EMOTIONAL HEALTH PROBLEMS:**  5、During the past 54 weeks, have you had any of the following problems with your work or other regular daily activities a result of any emotional problems (such as feeling depressed or anxious)?  （1）Cut down the amount of time you spent on work or other activities  ①Yes ②No   1. Accomplished less than you would like 2. ①Yes ②No   （3）Didn’t do work or other activities as carefully as usual  ①Yes ②No  **SOCIAL ACTIVITIES:**  6.Emotional problems interfered with your normal social activities with family, friends, neighbors, or groups?  ①Not at all ②Slightly ③Moderately ④Severe ⑤Very severe  7.During the past 4 weeks, how much did pain interfere with your normal work (including both work outside the home and housework?  ①Not at all ②A little bit ③Moderately ④Quite a bit ⑤Extremely   1. In the past 4 weeks, has your body pain affected your work and housework?   ①Not at all ②Slightly ③Moderately ④Severe ⑤Very severe | | | |
| **ENERGY AND EMOTIONS**  9.The following questions are about your own feelings in the past month. What is your situation like what you said about each question?  （1）Do you feel that life is fulfilling:  ①All the time ②Most of the time ③More time ④Part of the time ⑤Small part of the time ⑥No such feeling   1. You are a sensitive person:   ①All the time ②Most of the time ③More time ④Part of the time ⑤Small part of the time ⑥No such feeling  （3）Your mood is very bad, nothing can make you happy:  ①All the time ②Most of the time ③More time ④Part of the time ⑤Small part of the time ⑥No such feeling   1. You are calm: 2. ①All the time ②Most of the time ③More time ④Part of the time ⑤Small part of the time ⑥No such feeling   （5）You are full of energy to do things:  ①All the time ②Most of the time ③More time ④Part of the time ⑤Small part of the time ⑥No such feeling  （6）Your mood is low:  ①All the time ②Most of the time ③More time ④Part of the time ⑤Small part of the time ⑥No such feeling  （7）You feel exhausted:  ①All the time ②Most of the time ③More time ④Part of the time ⑤Small part of the time ⑥No such feeling  （8）You are a happy person:  ①All the time ②Most of the time ③More time ④Part of the time ⑤Small part of the time ⑥No such feeling  （9）You feel bored:  ①All the time ②Most of the time ③More time ④Part of the time ⑤Small part of the time ⑥No such feeling  (10)Unhealthy affects your social activities (such as visiting relatives and friends):  ①All the time ②Most of the time ③More time ④Part of the time ⑤Small part of the time ⑥No such feeling | | | |
| **General health**  10. Please look at each of the following questions. Which answer best suits your situation?  （1） I seem to get sick more easily than others:  ①Absolutely correct ②Mostly correct ③Uncertainty ④Most wrong ⑤Absolutely wrong   1. I am as healthy as the people around me:   ①Absolutely correct ②Mostly correct ③Uncertainty ④Most wrong ⑤Absolutely wrong  （3）I think my health is getting worse:  ①Absolutely correct ②Mostly correct ③Uncertainty ④Most wrong ⑤Absolutely wrong  （4）My health is very good:  ①Absolutely correct ②Mostly correct ③Uncertainty ④Most wrong ⑤Absolutely wrong | | | |
